# Supplementary material for: Patterns of Intron Gain and Loss in Fungi
Source: PLoS Biol. 2004 Nov 30;2(12):e422. doi: 10.1371/journal.pbio.0020422 (PMC532390; doi:10.1371/journal.pbio.0020422)
Supplement: Table S1 — Also available at http://genes.mit.edu/NielsenEtAl/. (4.3 MB ZIP). [file pbio.0020422.st001.zip › NielsenEtAl/html/520.html]

AN4870.1.NCU08972.1.MG01152.1.FG10254.1


```
 CLUSTAL W (1.82) Multiple Sequence Alignments - Introns Inserted


Sequence 1: MG01152.1	173 aa
Sequence 2: FG10254.1	157 aa
Sequence 3: NCU08972.1	184 aa
Sequence 4: AN4870.1	180 aa
Alignment Length: 184 aa
Number Identitical Residues: 42 aa
Alignment Score (without introns) 2950


MG01152.1 	-MDGC1---------ELLADFRDLQTWIA~AAPETPEDMDDYYSEGWAALRQCALDGHHI
NCU08972.1	MSDGL1NPLREARVAELLSDFRTLQYLIA~AVPTCPSNPDDYYTEGWAALRQCALDGQHI
FG10254.1 	-MDG-~----------------------S1AAPVDPVDTADYYTEGWAALRQCALDGQHI
AN4870.1  	MADGL1NQARALRVAELMNDYRTLLVHIN~QLN-VPVPPADQSEEGYRVLRECVAAANAL
          	  **  .       :.   .               *    *   **: .**:*.  .: :

MG01152.1 	LNCGADTSVPQVRG-GQEEQEKAELLQ~NHLDAFSRRHESHKIWLRQGAAQRWVAARFQI
NCU08972.1	LNCAADISIPCASGPGPDEQAKAELKQ~VLLDAYARRHEGQKIYLRQAAAQRWVGWRESV
FG10254.1 	LECGADTSVPNPPG-GEQEQMKAELKQ2VLLDAYSRRHEGQKIYLRQAAAQRWVEYRNQV
AN4870.1  	MASNYTPSPPPANT-SADEAEKAQLQR2VILDGSARRFQAHRIYLRTAAAKRWIIHRDGL
          	: .    * *     . :*  **:* :   **. :**.:.::*:** .**:**:  *  :

MG01152.1 	LQGRRPHAEHSAQLKACDRALRR0EYETITDEAVYNQLQASDIELGRWTGEDPSLRAVQR
NCU08972.1	VNGGGGGGGCRQEMEGMDGQLRA0ELANITDEAIYAELQANDIRCGRWVDEDPSLREVQR
FG10254.1 	LQGGVPNSRNQSQLRACDQQLRA0ELSAVVDEVIYNELKASDEQMGRWTAEDPSLRSVLR
AN4870.1  	LRGQRPGPQHAGPMKTISNTLRE0ELAQISNQHVFADLRAADVRAGHWLAEDPTLDTILS
          	:.*          :.  .  **  *   : :: :: :*:* * . *:*  ***:*  :  

MG01152.1 	WLRSRRRA
NCU08972.1	WIRSRSQC
FG10254.1 	WLRARR--
AN4870.1  	WIRSHP--
          	*:*::
```
